# Spending and Out-of-Pocket Prices for Brand-Name Drugs Among Commercially Insured Individuals in Massachusetts, 2015-2017

**DOI:** 10.1001/jamanetworkopen.2021.3252

**Published:** 2021-03-25

**Authors:** Micah B. Aaron, Anna D. Sinaiko

**Affiliations:** 1 Health Policy Department, Harvard University, Boston, Massachusetts; 2 Department of Health Policy and Management, Harvard T.H. Chan School of Public Health, Boston, Massachusetts

## Abstract

This cross-sectional study analyzes data on aggregate utilization and aggregate spending by patients and by pharmacy benefit managers to pharmacies for every brand-name drug prescription dispensed to all fully insured patients and some self-insured patients with commercial insurance in Massachusetts from 2015 to 2017.

## Introduction

In 2019, one-quarter of persons living in the United States younger than 65 years of age reported that it was “difficult” to afford their prescription drugs.^1^ High drug costs for patients have been associated with worse medication adherence—which may lead to poor health—and with poor financial outcomes for patients.^2^ Several policy proposals are considering forms of drug price regulation for the highest-priced drugs to slow the increase in drug spending. Understanding how these policies may affect the affordability of medications for patients requires evidence of actual patient out-of-pocket (OOP) prices,^3^ and understanding how prescription drug prices translate into OOP prices. Affordability for patients also depends on the increase in OOP prices over time. In this study, we investigated out-of-pocket prices for brand-name drugs, growth in these prices over time, and how these prices translated into patient spending.

## Methods

For this cross-sectional study, we obtained data on aggregate utilization (number of prescriptions, total days’ supply) and aggregate spending by patients and by pharmacy benefit managers to pharmacies for every brand-name drug prescription (excluding specialty drugs) dispensed to all fully insured patients (44.4% of commercial market in 2017) and some self-insured patients with commercial insurance in Massachusetts from 2015 to 2017.^4^ Data were derived from the Massachusetts All-Payer Claims Database and represented 1510 brand-name drugs and 8.45 million prescriptions. Analyses were conducted between February and October 2020 using the R statistical software (R Project for Statistical Computing). This cross-sectional study follows the Strengthening the Reporting of Observational Studies in Epidemiology (STROBE) reporting guideline. Given the use of deidentified data exclusively, the study was deemed not to require ethics board review based on the policy of the Office of Human Subjects Research Protections, National Institutes of Health, under the revised Common Rule. We analyzed publicly available, aggregated drug-year level data.

For each drug-year, we calculated the mean point-of-service price per 30-day prescription (ie, excluding rebates and discounts that may affect net spending),^5,6^ the mean patient OOP price per 30-day prescription, and the mean patient-paid proportion of point-of-service price (“patient share”). The point-of-service price is not the manufacturer list price; it is the negotiated reimbursement price paid to the pharmacy and is used to calculate patient cost sharing. For drugs not covered by one’s insurance plan (eg, those subject to a deductible), patients pay point-of-service price. We categorized drugs into quartiles based on point-of-service price. For drugs appearing in both 2015 and 2017 (66% of drugs), we measured the increase in OOP price using the compound annual growth rate.

## Results

While a large portion (42.7%) of patient spending was for drugs in the highest price quartile, more than half (53%) of patient spending was for drugs in the second and third price quartiles. In contrast, 63.9% of total spending was for drugs in the highest price quartile, and 33.0% was for drugs in the second and third quartiles (Figure). The OOP price for patients was more than 25% of the point-of-service price for 41.3% of drugs in the second price quartile, for 5.4% of drugs in the third quartile, and for 1.1% of the top quartile.

**Figure.**
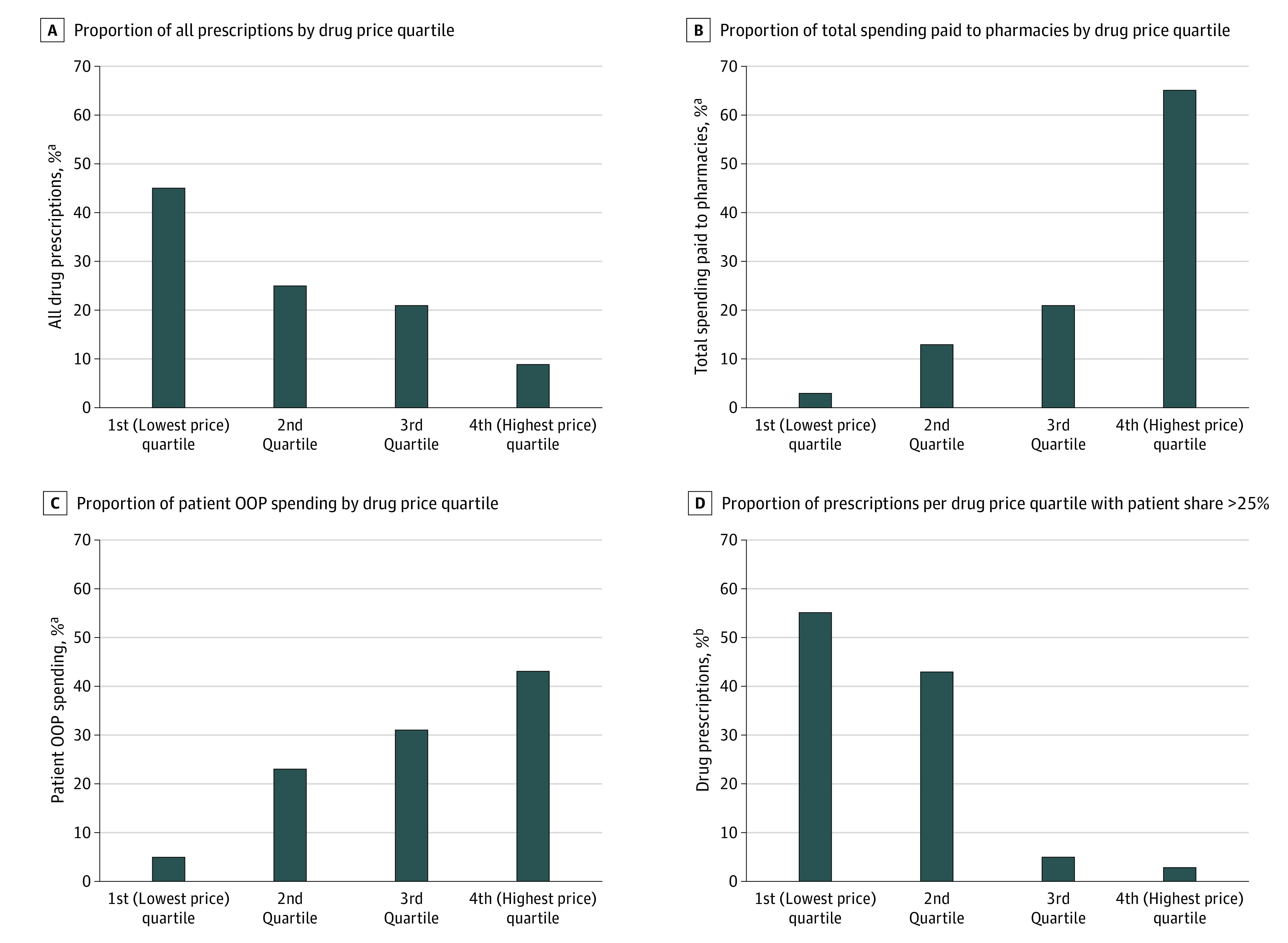
Proportion of Brand-Name Prescriptions, Proportion of Spending, and Variation in Patient Share of Price by Point-of-Service Drug Price Quartile Brand-name prescription drug use and spending data were obtained from the Center for Health Information and Analysis, based on pharmacy claims from mostly fully insured individuals from 11 commercial payers from the Massachusetts All-Payer Claims Database, 2015-2017. We included all drug-years in the data set with at least 30 prescriptions per year (3723 drug-year observations). All prices and spending were estimated net of inflation using the 2017 Consumer Price Index. OOP indicates out-of-pocket. ^a^Drugs assigned to quartile based on distribution of point-of-service price per 30-day fill in each year. In 2015, quartile 1 included point-of-service prices $0.01 to $71.40, quartile 2 included prices $71.50 to $268, quartile 3 included prices $268.01 to $693.70, and quartile 4 included prices of more than $693.70. In 2016, quartile 1 included point-of-service prices $0.01 to $84.00, quartile 2 included prices $84.10 to $301.50, quartile 3 included prices $301.60 to $839.70, and quartile 4 included prices of more than $839.70. In 2017, quartile 1 included point-of-service prices $0.01 to $91.63, quartile 2 included prices $91.64 to $331.89, quartile 3 included prices $331.90 to $931.87, and quartile 4 included prices of more than $931.87 ^b^Patient share is the mean OOP price per 30-day prescription per drug-year divided by the point-of-service price per 30-day prescription per drug-year.

Across drugs, the mean OOP price per 30-day fill was $45.46, and the mean increase in OOP price from 2015 to 2017 was 43.9% (Table). For the 20 drugs with the highest OOP prices, the mean OOP price per 30-day fill ranged from $194.89 to $1937.25, and the 2015 to 2017 increase in OOP price ranged from −28% to 140%.

**Table.** Top 20 Brand-Name Drugs by Mean Patient OOP Price, With Point-of-Service Price and 2015-2017 OOP Price Growth Rates^a^

Drug name	Therapeutic class	Price per 30-d fill		
		Mean OOP, $^b^	Annual growth rate in OOP, %^c^	Mean point-of-service, $^d^
All drugs	Not applicable	45.46	43.9	2286.50
Firazyr	Anti-inflammatory	1937.25	−21.8	429 892.46
Ovidrel	Hormones	470.82	−1.3	1353.32
Prepopik	Gastrointestinal	400.92	−23.7	1276.56
Suprep	Gastrointestinal	400.09	−27.8	888.36
Biltricide	Anti-infectives and miscellaneous	389.68	16.2	4765.80
Gynazole 1	Antifungals	336.08	−25.4	642.32
Osmoprep	Gastrointestinal	306.17	21.7	771.00
Clindesse	Antibiotics	285.13	41.9	545.68
Moviprep	Gastrointestinal	259.52	−27.8	545.90
Picato	Antineoplastics	259.31	−5.6	2988.10
Gonal-FRFF Redi-JECT	Hormones	259.13	13.3	16 678.37
Follistim AQ	Hormones	251.63	38.9	16 910.17
Dificid	Antibiotics	239.67	140.0	9129.80
Cetrotide	Hormones	239.30	14.9	5361.15
Epipen Jr 2-pak	Autonomic drugs	232.49	15.6	2301.15
Cleocin	Antibiotics	220.34	40.3	712.40
Auvi-Q	Autonomic drugs	207.06	−7.6	4186.88
Sklice	Skin Preparations	202.91	13.8	959.62
Tamiflu	Antivirals	201.68	16.4	606.96
Epipen 2-pak	Autonomic drugs	194.89	18.3	2048.32

Abbreviation: OOP, out-of-pocket.

^a^ Brand-name prescription drug use and spending data were obtained from the Center for Health Information and Analysis, based on pharmacy claims from mostly fully insured individuals from 11 commercial payers from Massachusetts (All-Payer Claims Database, 2015-2017). We included all drug-years in the data set with at least 30 prescriptions per year (n = 1510).

^b^ The mean OOP price per 30-day prescription equals the mean OOP price per 30-day fill per year, from 2015 to 2017.

^c^ Growth rate was measured using the compound annual growth rate, reported as a percentage.

^d^ The mean point-of-service price per 30-day prescription equals the mean point-of-service price per 30-day fill per year, from 2015 to 2017.

## Discussion

Because insurance covers very high shares of the point-of-service price for the most expensive drugs to avoid exposing patients to excessive financial risk, a large portion of patient OOP spending was for drugs with moderate prices (eg, second and third quartile) and not just for drugs with high (eg, top quartile) point-of-service prices. The OOP prices increased 43.9% from 2015 to 2017, a period when the increase in net drug prices was stable.^6^

The limitations of this cross-sectional study include that we cannot observe whether patient cost sharing is offset by coupons or copay assistance, and we cannot distinguish between when patients pay consistent OOP prices and when patients pay higher OOP prices at first and then reach their out-of-pocket maximum. Because the point-of-service price does not include rebates, our measure of patient share understates the fraction of drug cost (eg, net price) paid by patients. These findings may not be generalizable outside Massachusetts, where health insurance may be more comprehensive.

The findings of this study suggest that a policy to reduce the highest drug prices may lead to lower total spending and lower health insurance premiums, but it may not necessarily address the high OOP prices paid by many patients. Monitoring and slowing the increase in OOP spending will be important to improve patient affordability and access to prescription medications.
